# Role of the Macrophage Migration Inhibitory Factor (MIF) in the survival of first trimester human placenta under induced stress conditions

**DOI:** 10.1038/s41598-018-29797-6

**Published:** 2018-08-14

**Authors:** Francesca Ietta, Eloisa Amália Vieira Ferro, Estela Bevilacqua, Linda Benincasa, Emanuela Maioli, Luana Paulesu

**Affiliations:** 1 Department of Life Sciences, University of Siena, Via A. Moro 4, 53100 Siena, Italy; 2 Laboratory of Immunophysiology of Reproduction, Institute of Biomedical Sciences, Federal University of Uberlândia, Av. Pará 1720, 38405320 Uberlândia, Brazil; 3 Department of Cell and Developmental Biology, Institute of Biomedical Sciences, University of São Paulo, Av. Prof Lineu Prestes 1524, 05508-900 São Paulo, Brazil

## Abstract

Macrophage Migration Inhibitory Factor (MIF) is a multifunctional molecule highly secreted by human placenta mainly in the early phases of pregnancy. Studies in different cells show that MIF is a pro-survival factor by binding to its receptor CD74. By using the *in vitro* model of placental explants from first trimester pregnancy, we investigated the role of MIF in the survival of placental cells under induced stress conditions that promote apoptosis or mimic the hypoxia/re-oxygenation (H/R) injury that placenta could suffer *in vivo*. We demonstrated that recombinant MIF (rMIF) treatment was able to reduce caspase-3 activation when cultures were challenged with the apoptosis-inducer Carbonyl cyanide 4-(trifluoromethoxy)phenylhydrazone (FCCP) while, in the cultures exposed to H/R, the treatment with rMIF did not show any effect. However, a significant increase in caspase-3 and caspase-8 activation was found when H/R-exposed cultures, were treated with anti-MIF or anti-CD74 antibody. We also observed that under H/R, a significant amount of endogenous MIF was released into the medium, which could account for the lack of effect of rMIF added to the cultures. Our results demonstrate for the first time that the MIF/CD74 axis contributes to maintain trophoblast homeostasis, by preventing abnormal apoptotic death.

## Introduction

The placenta, the organ interposed between the mother and the foetus, performs critical functions for embryo growth i.e. the supply of nutrients, the transport of gases, ions, water and waste products^1^.

The placenta has also the ability to perform proper adaptive responses to various types of stressors that could negatively influence foetal growth thus playing a major role in optimizing the embryo/foetal development^2^.

One of the molecules highly present in the placenta is the cytokine Macrophage Migration Inhibitory factor (MIF)^3,4^, an evolutionarily conserved cytokine that is ubiquitously expressed by a variety of cells and tissues^5^. Originally described as a soluble factor produced by activated lymphocytes^6^, MIF is today recognized to exert functions in several physiological processes including the support of cell proliferation/ survival and prevention of cellular senescence^7,8^.

Various studies in different cell types, show that the pro-survival role of MIF is achieved through several distinct mechanisms, such as metabolic activation, autophagy induction, apoptosis suppression and anti-oxidative responses^9–12^.

MIF-triggered signals are mediated by its interaction with CD74, a single-pass type II membrane protein that is also known as the MHC class II chaperone invariant chain^13^. The MIF/CD74 axis has been considered the primary responsible for cell survival responses in the initial stages of tissue injury^12^.

The expression of MIF in placental tissues is particularly high during the early stages of pregnancy both in mice and humans^3,14^. The secretion of MIF by placental cells *in vitro* is increased by low oxygen values, comparable to those occurring in the early stages of pregnancy^3^. The levels of MIF in the intervillous space are reportedly higher than in maternal and cord blood and become even higher in consequence of placental insults such as malaria infection^15^. Altogether, the data on MIF in human pregnancy suggest that MIF is a key molecule in the placental response to endogenous and exogenous harmful stimuli.

Despite the number of studies on MIF in pregnancy^16,17^, only little is known about its role at the maternal-foetal interface. It has been demonstrated that trophoblast MIF reduces the cytotoxicity of human decidual NK cells^18^. Studies in mice have shown that recombinant MIF, *via* its interaction with CD74 receptor, sustains decidual cell survival *in vitro* by interfering with the fate of these cells when subjected to pro-apoptotic stimuli^19^.

In view of the above evidence, we suggest that MIF exerts its action also in the placenta and, in particular, we hypothesize that MIF protects trophoblast from apoptosis, a crucial cellular event in the earlier stages of pregnancy that could become harmful if not properly regulated^20^.

To verify the hypothesis, we investigated the role of exogenous and endogenous MIF and that of its receptor the CD74 in human placental explants at first trimester pregnancy, subjected to pro-apoptotic stimuli.

## Results

### CD74 expression in first trimester placental tissues and interaction with MIF

**Figure 1:**
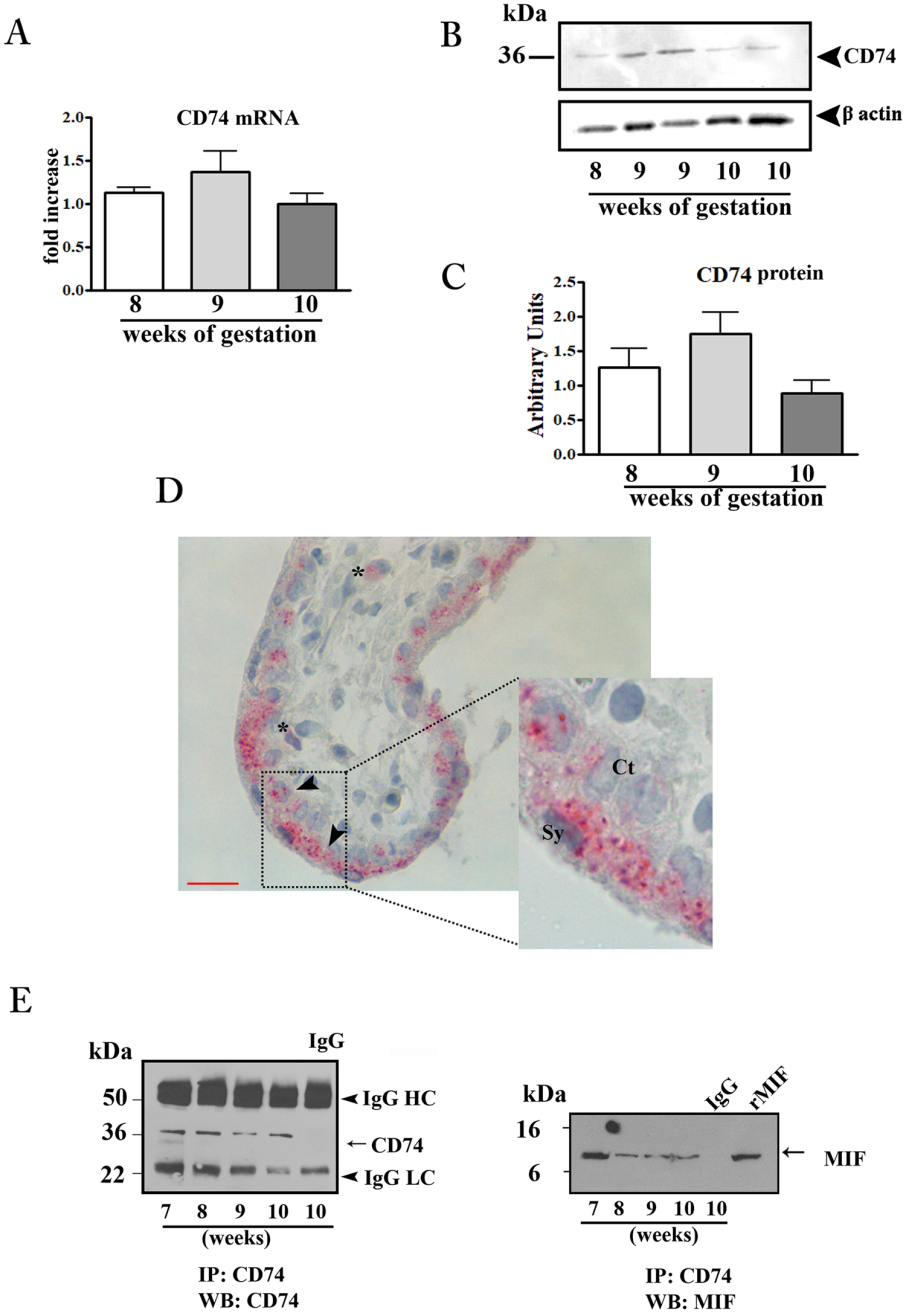
CD74 expression in first trimester placental tissues and interaction with MIF. CD74 mRNA expression assessed by qRT-PCR. (**A**) CD74 mRNA levels were normalized to those of 18S and expressed as fold increase relative to 8 weeks placental tissue selected as calibrator sample. Representative western blot (**B**) and densitometric analysis (**C**) in placental tissues at different weeks of gestation (n = 5 for each week). (**D**) Representative immunohistochemical analysis of CD74 in placenta at 9 weeks of gestation. Slides were counterstained with Mayer’s haematoxylin. Reddish staining represents positive immunoreactivity for CD74. Arrow-head indicates villous trophoblasts; asterisk marks the mesenchymal cells. Ct: cytotrophoblast; Sy: syncytiotrophoblast. Bar = 25 μm. (**E**) Representative immunoprecipitation (IP) of CD74 in placental tissues from 7 to −10 weeks of gestation followed by western blot (WB) for CD74 (left panel) and MIF (right panel). IgG: isotype control; rMIF: recombinant MIF.

In order to verify the sensitivity of placenta to MIF action we evaluated the CD74 expression in placental tissues throughout the first trimester of pregnancy. Both CD74 mRNA and protein were detected in first trimester placenta with a peak, although not significant, at 9 weeks of gestation (Fig. 1A–C).

Immunohistochemical analysis showed a dotted staining in trophoblast cells, both in the cytotrophoblast and syncytiotrophoblast layers and to a lesser extent in some mesenchymal cells (Figs 1D and 7S).

Immunoprecipitation experiments with anti CD74 antibody, followed by SDS-PAGE and immunoblotting against human MIF, revealed the presence of a positive band at 12 kDa co-migrating with the rMIF, showing an effective interaction between CD74 and MIF (Fig. 1E right panel). As a proof of this, CD74 immunoprecipitated samples, subjected to western blotting for CD74, showed the presence of a positive band at 37 kDa; no bands were obtained when lysates were incubated with normal isotype control IgG (Fig. 1E left panel).

### Induction of MIF release by hypoxia/re-oxygenation (H/R) in placental explant cultures

To evaluate the impact of apoptosis stimulation on placental MIF we pursued two approaches: (1) exposure of explants cultures to FCCP, a well known apoptosis inducer; (2) exposure of the explants cultures to H/R, a putative pro apoptotic *stimulus* that placenta might encounter *in vivo*.

**Figure 2:**
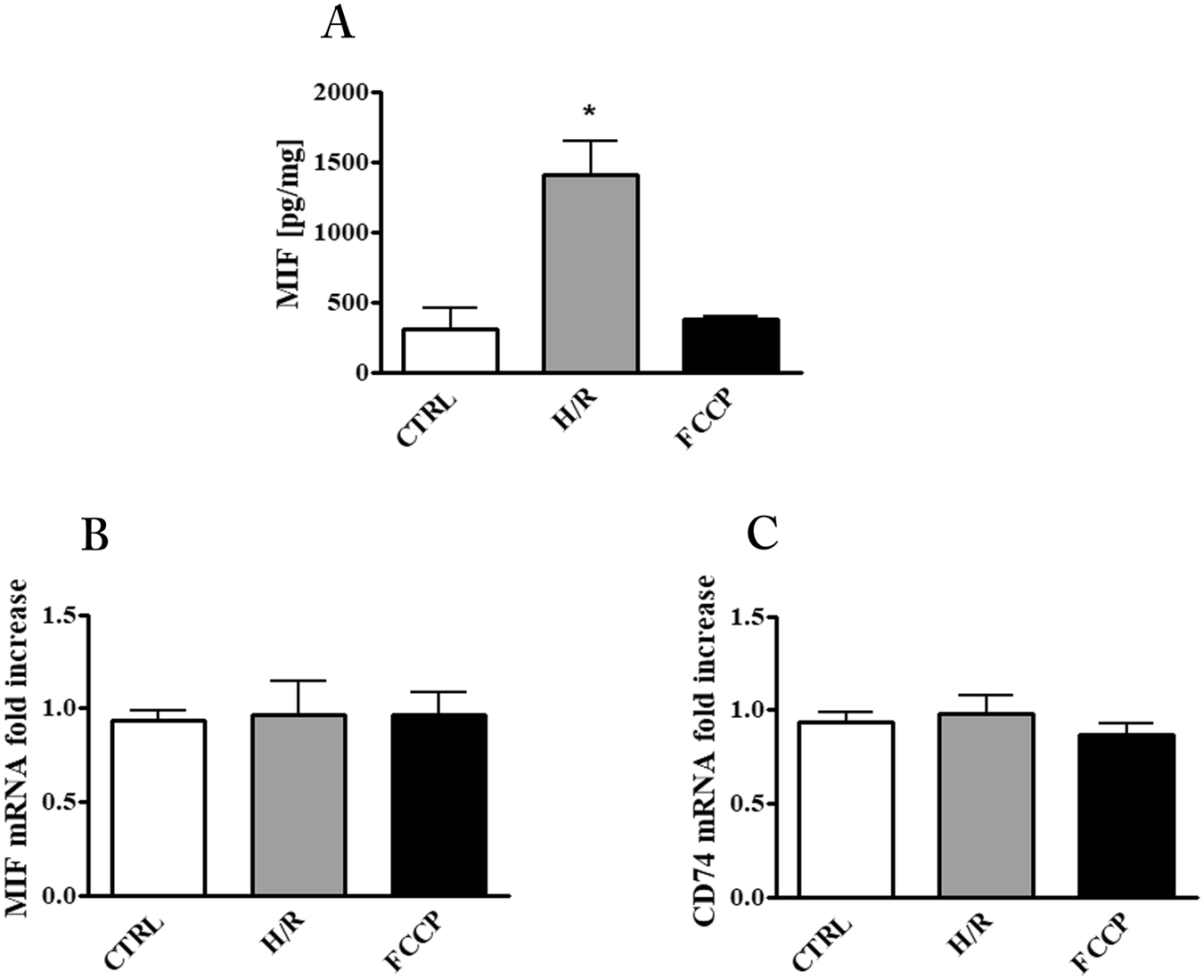
MIF and CD74 in placental explant cultures after exposure to hypoxia/re-oxygenation (H/R) and FCCP. (**A**) MIF concentration in culture supernatants quantified by ELISA assay and expressed as pg/mg (means ± SEM) of total explant tissue proteins. MIF (**B**) and CD74 (**C**) mRNA expression level assessed by qRT-PCR analysis. Results were normalized to those of 18 S and expressed as fold increase relative to control cultures. *p < 0.05 (n = 3 placentae at 8–9 weeks). CTRL: control explant cultures**;** H/R: explant cultures exposed to hypoxia/re-oxygenation; FCCP: explant cultures exposed to 10 µM Carbonyl cyanide 4-(trifluoromethoxy)phenylhydrazone.

Immunoassay for MIF in culture supernatants showed that H/R exposure produced a four-fold increase with respect to control cultures while, the FCCP treatment did not produce any change with respect to controls (Fig. 2A). In order to examine if the increased release of MIF by H/R was due to a cytotoxic effect of this specific treatment, LDH activity in the medium was analyzed. As shown in Table 1, the H/R protocol did not cause any change in LDH activity indicating that no appreciable cell damage occurred. Quantitative analysis of MIF and CD74 messengers showed no changes with any treatment (Fig. 2B and C). This finding indicates that the observed increase of MIF in supernatants might be due to its enhanced release from cellular stores or, alternatively, to an increased RNA translation/stabilisation.

**Table 1:** Placental explant cultures viability.

Sample	LDH
	Activity (OD/mg of total explant tissue proteins) (mean ± S.D)
CTRL	0.0625 ± 0.02
H/R	0.059 ± 0.023
FCCP	0.065 ± 0.025
Triton X100	0.253 ± 0.035

### rMIF protects placental explants from FCCP-induced apoptosis

Caspase-3 is the principal effector caspase in the execution of apoptotic cell death. In FCCP-induced apoptosis (intrinsic apoptosis pathway)^21^, caspase-3 is cleaved by activated caspase-9.

**Figure 3:**
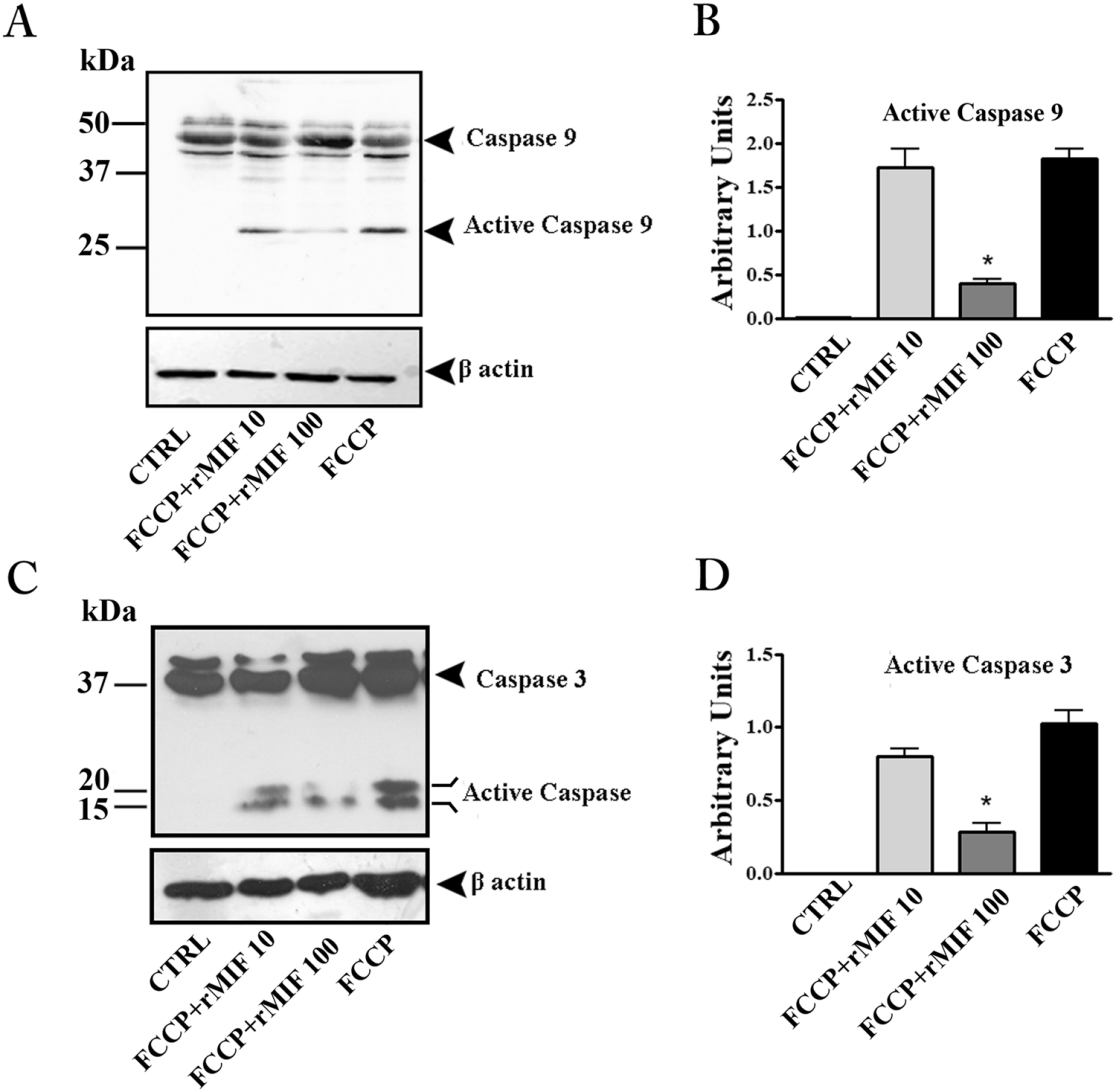
rMIF protects placental explants from FCCP-induced apoptosis. Representative western blots for caspase-9 (**A**) and caspase-3 (**C**) and their associated densitometric analyses (**B** and **D**) in placenta explant cultures treated with FCCP (10 µM), FCCP plus rMIF (10 and 100 ng/mL) or medium alone (CTRL). *p < 0.05 (n = 4 placentae at 8–9 weeks).

The western blotting analysis showed a substantial presence of cleaved forms of caspase-9 and -3 in explant cultures exposed to FCCP, demonstrating that the basic apoptotic machinery was intact in the cultures (Fig. 3). Treatment with rMIF reduced the activation of both caspases, with a statistically significant effect at 100 ng/mL, thus showing a clear anti-apoptotic effect (Fig. 3A–D).

### Inhibition of endogenous MIF activity triggers apoptosis in H/R exposed placental explants

**Figure 4:**
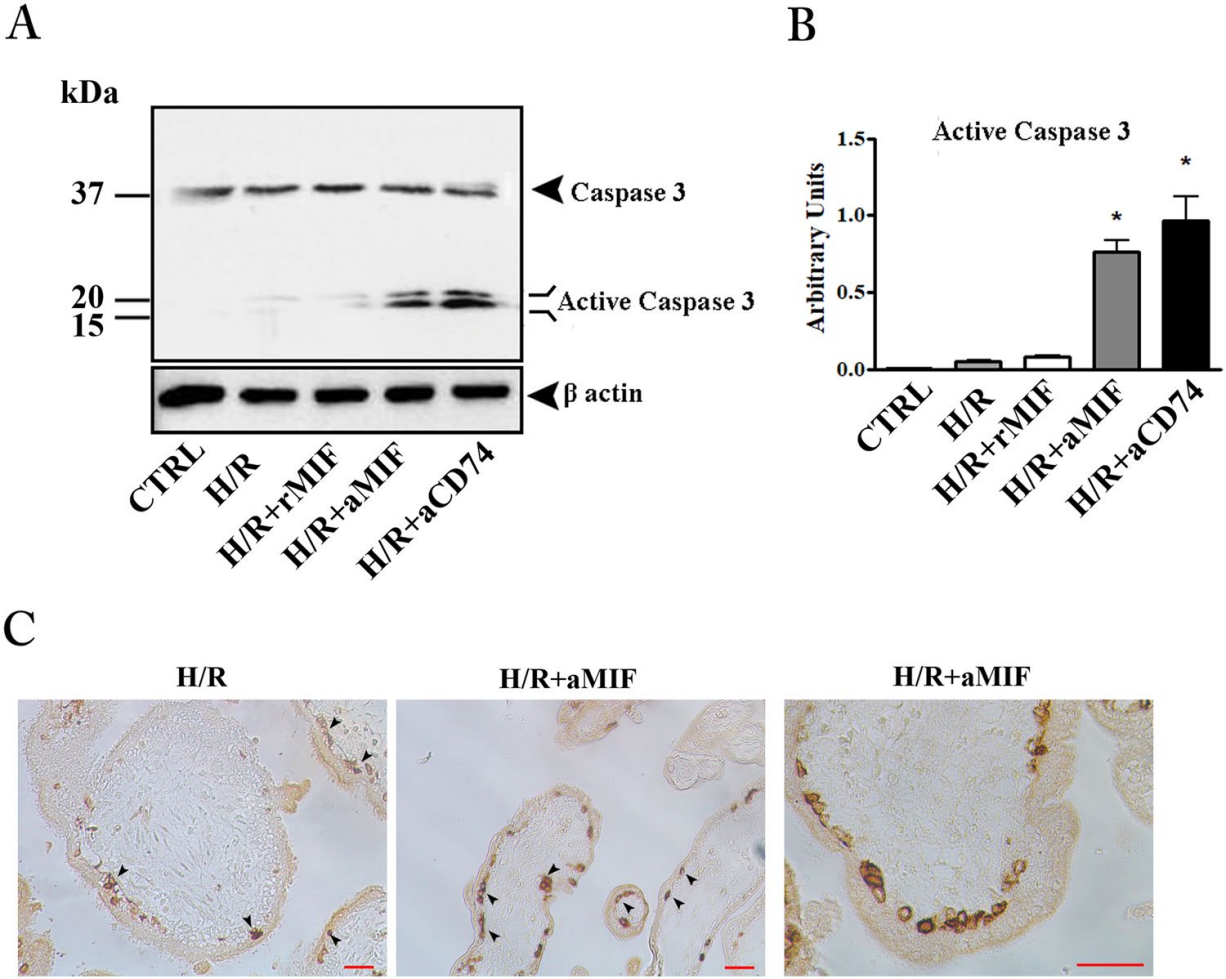
Inhibition of endogenous MIF triggers apoptosis in hypoxia/re-oxygenation (H/R) exposed placental explants. Representative western blot for caspase-3 (**A**) and the associated densitometric analysis (**B**) in explant cultures exposed to H/R. CTRL: control explant cultures**;** H/R + rMIF at 100 ng/mL (H/R + MIF); H/R + anti-human MIF at 10 µg/mL (H/R + aMIF) or anti-human CD74 at 5 µg/mL (H/R + aCD74). *p < 0.05 (n = 4 placentae at 8–10 weeks). (**C**) Representative immunohistochemical analysis of M30 in placental explants exposed to H/R and H/R + aMIF. Positive immunoreactivity is revealed by brownish colour. Bar = 25 μm.

Unlike FCCP treatment, H/R showed only faint bands corresponding to the active form of caspase-3 and addition of 100 ng/ml rMIF did not have any effect (Fig. 4). On the other hand, the blockage of MIF/CD74 interaction with anti-MIF or anti-CD74 antibodies added to the cultures, led to a significant caspase-3 activation (Fig. 4A and B). To gain insight into the importance of the MIF/CD74 axis in the protection against apoptosis, we analyzed explants cultures (exposed to H/R or H/R plus anti-MIF antibody) by histochemistry for M30 antigen, a specific marker for trophoblast apoptosis^22^. Only few M30 positive cells were observed in villous cytotrophoblasts in sections from explant cultures exposed solely to H/R (Fig. 4C left panel). A clear increase in the number of positive cells was shown in explants cultures exposed to H/R plus anti-MIF antibody (Fig. 4C, middle and right panels).

### H/R-apoptosis is via caspase-8 in placenta explants

In order to examine the apoptotic pathway triggered by H/R treatment in human placenta, we evaluated the activation status of caspase-8 and caspase-9. Indeed, apoptosis can be executed through the mitochondria (intrinsic) or cell ligand–receptors (extrinsic) pathways. Since both pathways converge on caspase-3, discrimination between the two was done by assessing activation of caspase-9 and caspase-8 for the intrinsic and the extrinsic pathway, respectively.

**Figure 5:**
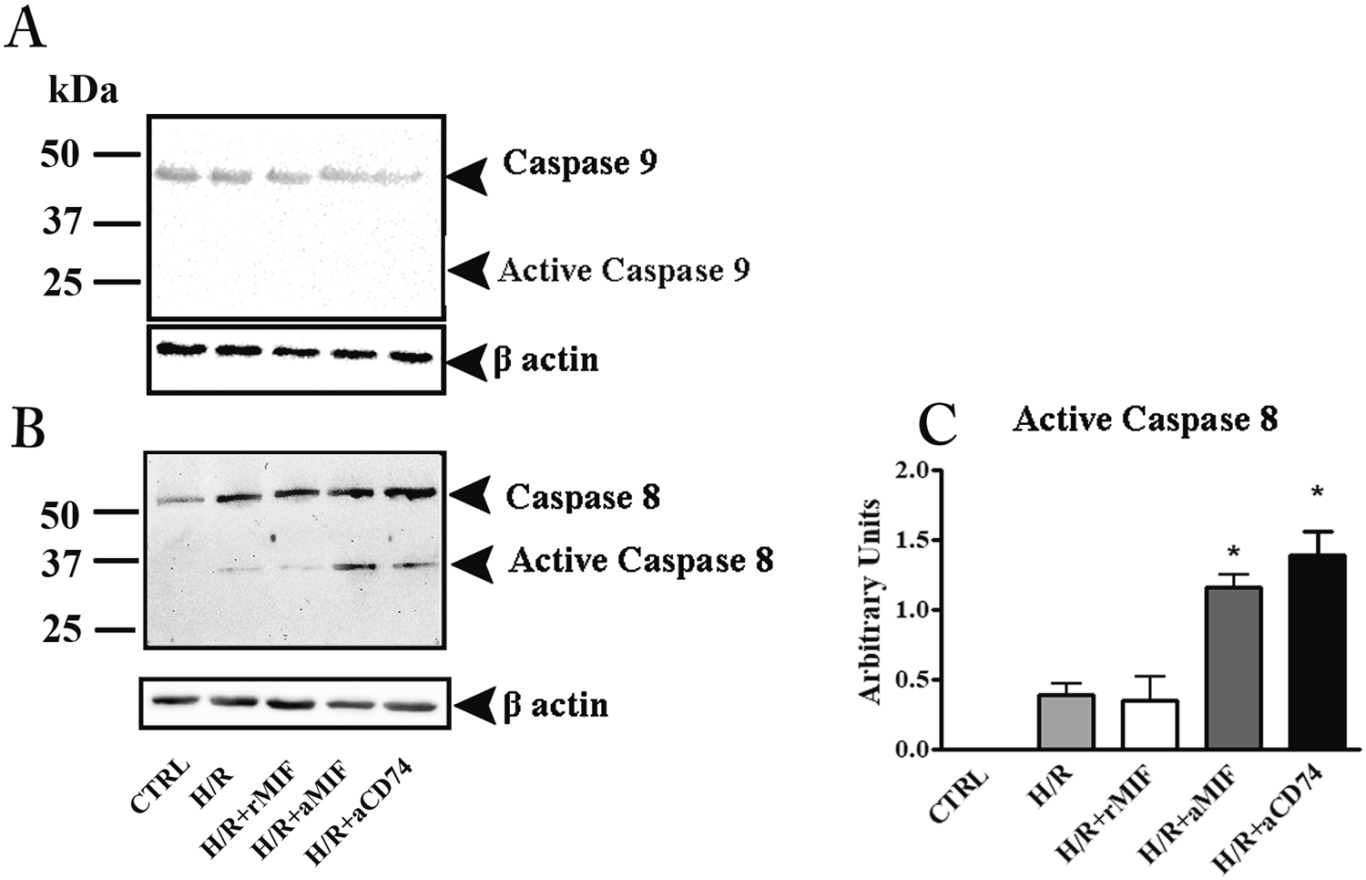
Hypoxia/re-oxygenation (H/R) apoptosis is via caspase-8 in placenta explants. Representative western blots for caspase-9 (**A**) and caspase-8 and the associated densitometric analysis (**B** and **C**) in explant cultures exposed to H/R. CTRL: control explant cultures**;** H/R + rMIF at 100 ng/mL (H/R + MIF); H/R + anti-human MIF antibodies at 10 µg/mL (H/R + aMIF) or anti-human CD74 antibodies at 5 µg/mL (H/R + aCD74). *p < 0.05 (n = 4 placentae at 8–10 weeks).

H/R exposure did not produce any expression of active caspase-9 fragment with any treatment (rMIF, anti MIF or anti CD74) (Fig. 5A) while, a band, although slight, was observed for caspase-8 in H/R with or without rMIF (Fig. 5B). As for caspase-3, the inhibition of MIF, or its activity by anti-MIF or anti-CD74, induced a marked increase in the expression of active caspase-8 fragments (Fig. 5B and C). To assess whether the various treatments directly induced apoptosis independently of H/R exposure, explants cultures, maintained at 20% O_2_, were treated with rMIF (100 ng/mL), anti-human MIF (10 µg/mL), anti-human CD74 (5 µg/mL) or normal isotype IgG antibody (10 µg/mL). The various treatments had no detectable effect on caspase-3 nor on caspase-9 (Fig. 8SA and B), while a clear activation of caspase-8 was shown in explants treated with anti −MIF and −CD74 antibody (Fig. 8SC).

## Discussion

MIF is a pluripotent cytokine with important functions as a pro-survival molecule in the response to environmental challenges such as hypoxia, hypoxia/re-oxygenation, starvation and inflammation in various cells and tissues^12,23,24^. However, the specific role of MIF in placenta tissues has never been explored.

In the current study, we identified MIF as a factor secreted by human placenta able to promote trophoblast survival. We showed that interaction of MIF with its receptor CD74 is a major mechanism through which MIF promotes trophoblast survival and suppresses apoptosis.

In our study, we applied a hypoxia/re-oxygenation (H/R) protocol mimicking what the placenta might meet *in vivo* in pathological circumstances^25^. Premature villous exposure to high levels of oxygen as well as hypoxia-reoxygenation injury have been implicated in complications of pregnancy i.e. miscarriage and pre-eclampsia^25,26^.

This study, performed on human placental explants from eight-ten weeks of pregnancy, showed that H/R condition increased release of MIF in the culture medium and that inhibition of MIF, or MIF activity by anti-MIF or anti-CD74, resulted in an induction of apoptosis. On the other hand, treatment with rMIF did not have any influence on apoptosis-induced H/R.

The data suggest that an appropriate placental secretion of MIF in response to H/R condition is essential to sensitize the cells against the death-inducing effects.

Nevertheless, the addition of rMIF, even at high concentration, to the cultures exposed to H/R did not have any influence on apoptosis resistance.

This result, albeit surprising, is in agreement with the study by Arcuri *et al*., reporting that while inhibition of placenta-secreted MIF increased uterine NK cell cytolytic activity, rMIF had only a slight inhibitory effect^18^.

The irrelevance of the rMIF addition in our model suggests that the high release of MIF by placenta under H/R is largely sufficient to prevent exaggerate apoptosis and no further protection is appreciable by adding exogenous MIF.

At support of this conclusion, we found that placental explants exposed to the apoptotic-inducer FCCP, which did not stimulate MIF release, strongly responded to the addition of rMIF, by reducing their apoptotic events.

It is important to underlie that H/R and FCCP use different pathways in the execution of apoptosis. It is known that FCCP induces mitochondrial damage (intrinsic apoptosis pathway)^27^ and the treatment with rMIF inhibits the mitochondrial release of cytochrome c and Smac thus preserving intact this cytoplasmic organelle in granulocytes^28^. As concerning H/R, a number of reports showed that the activation of apoptosis is via the intrinsic pathway with the involvement of the mitochondria^29–31^. However, the mechanisms behind H/R induced apoptosis are complex and can involve the intrinsic and/or extrinsic pathway depending on the cell type and the duration of stress^32^.

In our study we found that the intrinsic pathway was not affected by the H/R protocol as shown by the unmodified status of caspase-9, a marker of mitochondrial damage. On the other hand, caspase-8, a marker of the membrane-mediated extrinsic apoptosis pathway, was activated in H/R placental cultures particularly after treatment with anti-CD74 antibodies.

As shown by Berkova *et al*. in lymphoma cells, internalization of CD74, following the binding to MIF, results in (1) decreased membrane levels of Fas receptor, a major initiator of the apoptosis via the extrinsic pathway and (2) reduced sensitivity of the cells to pro-apoptotic stimuli^27^.

Similar mechanisms might occur in placenta because of the importance of Fas receptor in trophoblast apoptosis^33,34^. Further studies are needed to verify the internalization of MIF upon interaction of CD74 and the relation with membrane expression of Fas ligand in trophoblast.

Overall, we provided evidence that the cytokine MIF is a novel anti-apoptotic factor in placenta regardless the apoptosis pathway.

Our group previously demonstrated that MIF is highly expressed by human placenta particularly in the villous cytotrophoblast forming the internal proliferative layer of chorionic villi^3^. The current study showing the expression of MIF receptor CD74 in the villous trophoblast, at both messenger and protein level is suggestive of an autocrine/paracrine action of MIF in placenta.

Placenta throughout its entire existence is exposed to a variety of environmental stressors and its adaptive response is fundamental for pregnancy and foetal health^35^. This supports the notion that placenta possesses endogenous inhibitor(s) that de-sensitize itself to certain dangerous stimuli.

While the nature of this extraordinary resistance is likely to be multifactorial, a number of evidence suggests the cytokine MIF is a fundamental player.

Indeed, the consistent presence of MIF in human placenta and its potential to act at the maternal-foetal interface appear fundamental for the outcome of pregnancy. Yamada *et al*. showed that decreased maternal MIF serum levels are associated with recurrent miscarriage^36^. More recently, in a prospective study to investigate the status of maternal MIF serum levels in women who subsequently developed pre-eclampsia, we found a decrease in maternal MIF serum levels during the first or the early second trimester of pregnancy in comparison to normal pregnancies^37^. Of note, MIF action appears also important, Przybyl *et al*. recently showed that CD74 is one of the most down-regulated genes in placentae from women affected by pre-eclampsia^38^.

**Figure 6:**
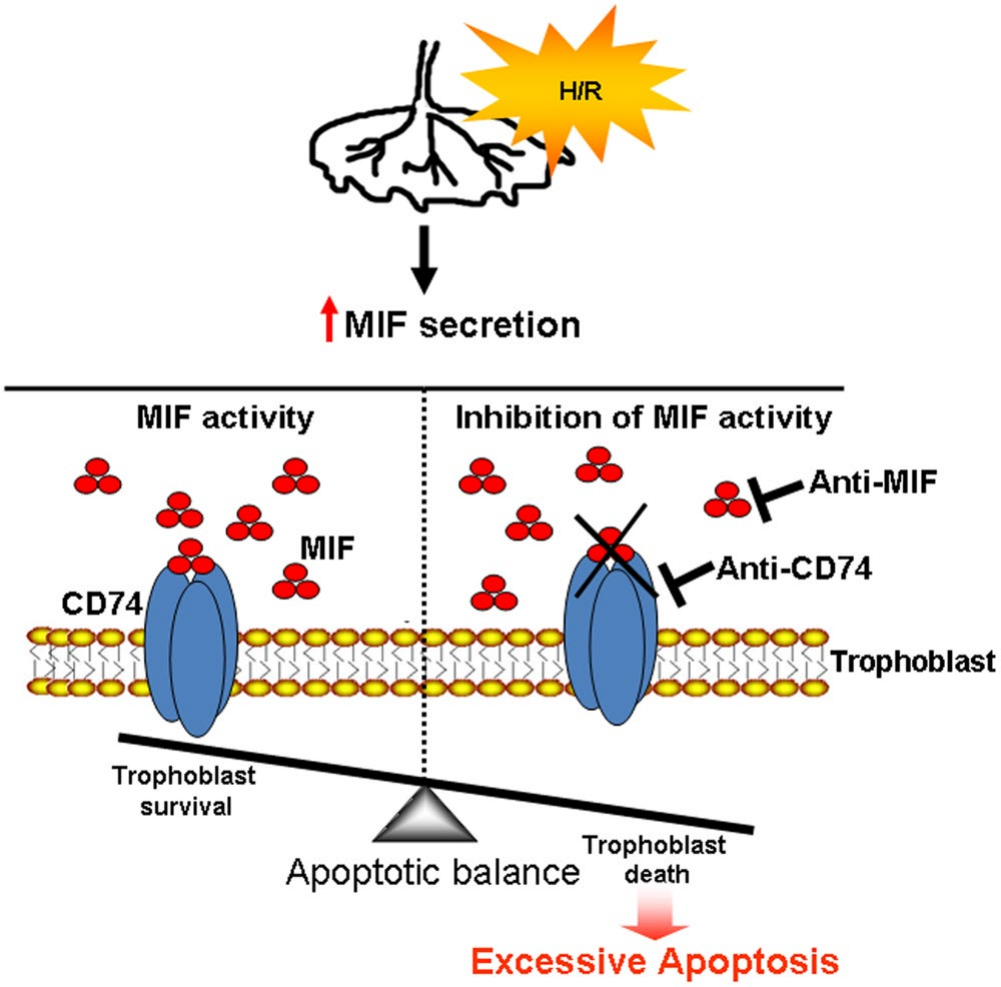
Schematic representation of the role of MIF in the protection of first trimester human placenta against hypoxia/re-oxygenation insults. Upon insults (hypoxia/re-oxygenation, H/R), the placenta increases the release of MIF that, binding to its membrane receptor CD74, protects trophoblast against excessive apoptosis (trophoblast survival). Inhibition of MIF or its binding to CD74 triggers apoptosis (trophoblast death).

Taken together, our data clearly show that the MIF/CD74 axis acts to prevent excessive apoptosis and to maintain trophoblast homeostasis in physiological pregnancy (see model depicted in Fig. 6)

## Materials

### Ethical approval

This study was conducted according to the Italian law on privacy and to the principles outlined in the Declaration of Helsinki. Human placenta tissues collection has already obtained the approval of Ethics Committee of University of Siena and Azienda Ospedaliera Senese (VITRO-RIP 2013). All patients enrolled provided written informed consent for the collection of samples and subsequent analysis.

### Tissues Collection

First trimester placental tissues (8–10 weeks of gestation, n = 25) were obtained at the Obstetrics and Gynecology Division (Campostaggia Hospital, Siena, Italy) after elective termination of pregnancy by dilatation and curettage. All samples immediately after collection were in part snap-frozen and stored at −80 °C and in part, formalin fixed for subsequently analyses. The remnant tissue was processed for chorionic villous explant cultures.

### Human chorionic villous explants cultures

Explant cultures were processed within 2 hours of collection. After several washes with sterile phosphate-buffered saline (PBS), villous trees were dissected under a stereomicroscope and explants cultures were performed as described by Caniggia *et al*.^39^. Explants were cultured in DMEM F12 (phenol red and serum free) plus L-glutamine (Gibco, Invitrogen, Basel, CH) and 1% antibiotics (penicillin-streptomycin) (Sigma Chemical Co. St. Louis, MO) and incubated overnight at 37 °C in 5% CO_2_ −95% air (standard conditions). Medium was then replaced with fresh one containing the various treatments.

In a first set of experiments, the medium was added with the apoptosis inducer, Carbonyl cyanide 4-(trifluoromethoxy)phenylhydrazone (FCCP) (Sigma Chemical Co.) (10 µM), in presence or in absence of recombinant MIF (rMIF) (R&D Systems, Abingdon, UK) (10 or 100 ng/mL) and incubated for further 3 hours, under standard conditions. Parallel explant cultures were maintained in medium alone (controls: CTRL).

In a second set of experiments, after replacement with fresh medium (phenol red and serum free DMEM F12, L-glutamine and 1% antibiotics), explant cultures were exposed to hypoxia/re-oxygenation (H/R) treatment as described by Hung *et al*.^30^ with some modification. Specifically, from standard conditions corresponding to 20% O_2_, cultures were exposed to low-oxygen conditions (5% CO_2_–93% N_2_) corresponding to 2% O_2_ for 1 hour, then, to re-oxygenation (20% O_2_) for 2 hours. The 2% O_2_ condition was achieved using a humidified chamber (BioSpa Division; Milan, Italy) flushed for 5 minutes with the appropriate gas mixture. Alternatively, the replaced fresh medium contained rMIF (R&D Systems) at 100 ng/mL, anti-human CD74 (Santa Cruz Biotechnology, Santa Cruz, CA) at 5 µg/mL or anti-human MIF (R&D Systems) at 10 µg/mL and cultures were exposed to H/R treatment as above (2% O_2_, 1 hour, followed by 20% O_2_, 2 hours). Control explant cultures treated or not with rMIF (100 ng/mL), anti-human CD74 (5 µg/mL), anti-human MIF (10 µg/mL) and isotype matched IgG antibody (10 µg/mL) were left for all time of experiments at 20% O_2_.

At the end of experiments, explant cultures were collected and processed for protein and mRNA expression by western blot and quantitative real time PCR (qRT-PCR), others were processed for immunohistochemistry. The supernatants were centrifuged and used for lactate dehydrogenase activity (LDH) and MIF determination.

For each type of experiment, explant cultures were carried out from the same placenta and each experimental condition was set up in quadruplicate. A total of 15 placentae were used.

### RNA isolation and qRT-PCR

Total RNA was isolated from placental samples or chorionic villous explants using TRIzol (Invitrogen, Carlsbad, CA) according to the manufacturer’s instructions. One µg of total RNA was reverse transcribed using random hexamer and MultiScribe enzyme (Applied Biosystems Group, Foster City, CA). qRT-PCR reactions were run in the StepOne™ Real-Time PCR System instrument (Applied Biosystems Group) using TaqMan chemistry. Two µL cDNA in a final volume of 20 µL were amplified using the Fast Universal PCR Master Mix (2X) (Applied Biosystems Group). TaqMan probes and specific primers for CD74, MIF and ribosomal 18S, selected as housekeeping gene, were purchased from Applied Biosystems Group. Expression level of the selected genes was calculated by the 2^−ΔΔCt^ ^40^.

### Western Blot

Total proteins from placental samples or chorionic villous explants were obtained by mechanical homogenization in RIPA buffer (50 mM Tris-HCl, pH 7.5; 150 mM NaCl; 1% [vol/vol] Triton X-100; 1% [wt/vol] sodium deoxycholate; 0.1% [wt/vol] SDS; 1 mM Na_2_VO_3_; 25 mM NaF; and protease inhibitors cocktail [Sigma Chemical Co.]). Samples were centrifuged at 15000 × g at 4 °C for 15 minutes, and the supernatants collected for total protein determination.

Western blot analysis was performed using 50 μg of total protein lysates that were subjected to 10% or 12% SDS-PAGE gel. Proteins were electro-transferred onto PVDF membranes and then blocked in 4% non-fat dry milk in Tris-buffered saline pH 7.2 (TBS) containing 0.1% Tween 20 for 2 hours at room temperature. Membranes were then incubated with polyclonal antibodies anti-human CD74 (Santa Cruz Biotechnology), MIF (R&D Systems), Caspase-3 (R&D Systems), Caspase-9, Caspase-8, (Cell Signaling Technology, Boston, MA USA), and β-actin (Santa Cruz Biotechnology) at dilution of 1:1000 overnight at 4 °C. After that, membranes were washed and incubated for 60 min at room temperature with the appropriated horseradish peroxidase-conjugated IgG (Santa Cruz Biotechnology). The reaction was revealed using chemiluminescent reagent (BioRad Laboratories Inc., Cambridge, MA) and then membranes exposed on photographic film, or digitalized with CHEMI DOC Quantity One program (BioRad Laboratories Inc.).

### Immunoprecipitation

Proteins from placental tissues at various gestational age (n = 7; 4 samples at 8 weeks, 3 samples at 9 weeks) were diluted in RIPA buffer to 1 µg/µL and then pre-cleared with 75 μl of protein-G Sepharose matrix (25% matrix, 75% binding buffer) (Sigma Chemical Co.) for 30 min at room temperature, with constant rocking. Unspecific complexes were eliminated by centrifugation at 10000 × g at 4 °C for 5 minutes, the supernatants were retained on ice, and the pellet was discarded. Fifteen μg of rabbit anti-human CD74 (Santa Cruz Biotechnology) or rabbit IgG isotype control (Sigma Chemical Co.) were added to samples and gently mixed overnight at 4 °C on a rocker. Immuno-complexes were captured by adding 100 μl protein-G Sepharose matrix after incubation for 2 hours. Samples were then centrifuged at 10000 × g for 5 minutes, washed three times with 1 mL ice-cold PBS and finally re-suspended in SDS loading buffer, boiled for 5 minutes, centrifuged, and the CD74/MIF association analyzed by western blotting.

### Immunohistochemistry

Immunohistochemistry was performed on 5 μm thick sections of formalin-fixed, paraffin embedded first trimester placental tissues (n = 3) using avidin-biotinylated horseradish peroxidase macromolecular, according to the manufacturer’s instructions (Sigma Chemical Co.) and slides were counterstained or not using Mayer’s haematoxylin. Polyclonal goat anti-human CD74 (1: 100 dilutions; Santa Cruz Biotechnology) and monoclonal mouse anti-cytokeratin 18 neo-epitope (1: 50 dilutions, clone M30; Roche, Mannheim, Germany) were used as primary antibody. Secondary antibody (1: 500 dilutions) was biotinylated rabbit anti-goat or mouse IgG (DAKO, Glostrup, Denmark). The reaction was developed with fast red-naphthol (Sigma Chemical Co.) or DAB (Thermo Fisher Scientific; Fremont, CA) following the manufacturer’s instructions. Controls were performed by substituting the primary antibody by the appropriate normal isotype antibody.

### ELISA

MIF was measured in the supernatants of explant cultures using a sandwich ELISA as described in Ietta *et al*.^41^ applying anti-human MIF monoclonal antibody (2.0 µg/mL) as capture antibody and biotinylated goat anti-human MIF polyclonal antibody (200 ng/mL) as detection antibody from R&D Systems. The sensitivity limit of the assay is 18 pg/mL. Intra- and inter-assay coefficients of variation are 3.86% ± 0.95% and 9.14% ± 0.47%, respectively. Data were expressed as pg/mg after normalization to total proteins of the respective explant homogenate.

### Placenta Explant cultures viability assay

Effect of the FCCP or H/R treatment on viability of placental explant cultures was evaluated by measurement of LDH activity in the supernatants using Toxicology Assay Kit (Sigma Chemical Co.) according to the manufacturer’s protocol. Maximum LDH release was obtained by adding 0.2% Triton X-100 to placenta explant cultures, which was identified as positive control (total cell lysis). LDH values (optical units: OU) were normalized to total proteins of the respective explant homogenate.

### Statistical analysis

Densitometric values for Western blots analysis were performed using Quantity One software (BioRad Laboratories Inc). The data were graphically processed and analyzed with GraphPad Prism 4. Comparison between 2 groups was performed using two-tailed t-test. p < 0.05 was considered to be statistically significant.

## Electronic supplementary material

Supplementary materials
