# Supplementary material for: Role of the Macrophage Migration Inhibitory Factor (MIF) in the survival of first trimester human placenta under induced stress conditions
Source: Sci Rep. 2018 Aug 14;8:12150. doi: 10.1038/s41598-018-29797-6 (PMC6092320; doi:10.1038/s41598-018-29797-6)
Supplement: Supplementary file 1 — Supplementary materials [file 41598_2018_29797_MOESM1_ESM.doc]

**Supplementary dataset for**

**Role of the Macrophage Migration Inhibitory Factor (MIF) in the survival of first trimester human placenta under induced stress conditions**

Francesca Ietta^a*^, Eloisa Amália Vieira Ferro^b^, Estela Bevilacqua^c^, Linda Benincasa^a^, Emanuela Maioli^a^, Luana Paulesu^a^

^a^Department of Life Sciences, University of Siena, Via A. Moro 4, 53100 Siena Italy;

^b^Laboratory of Immunophysiology of Reproduction, Institute of Biomedical Sciences, Federal University of Uberlândia, Av. Pará 1720, 38405320 Uberlândia, Brazil;

^c^Department of Cell and Developmental Biology, Institute of Biomedical Sciences, University of São Paulo, Av. Prof Lineu Prestes 1524, 05508-900 São Paulo, Brazil.

*Corresponding author: francesca.ietta@unisi.it

**Supplementary figures online**


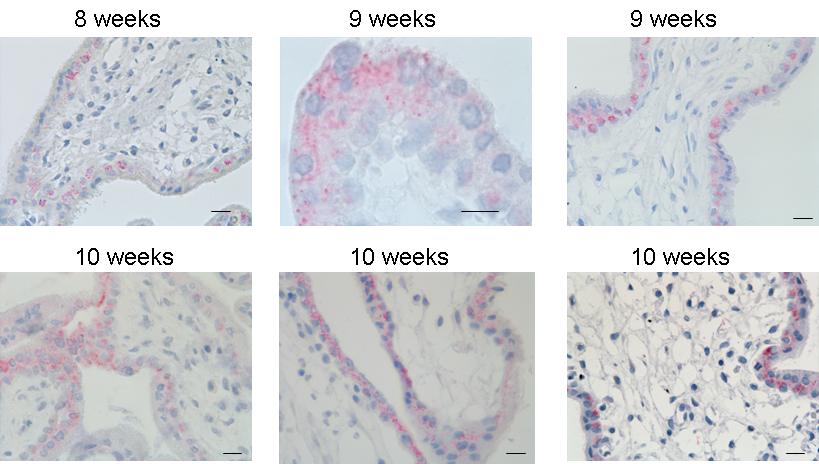


**Supplementary Figure 7S. CD74 localization in human placenta throughout gestation.** Representative histochemical analysis for CD74 in placental tissues at different gestational ages. Slides were counterstaining with Mayer’s haematoxylin. Reddish staining represents positive immunoreactivity for CD74. Bar = 25 μm.


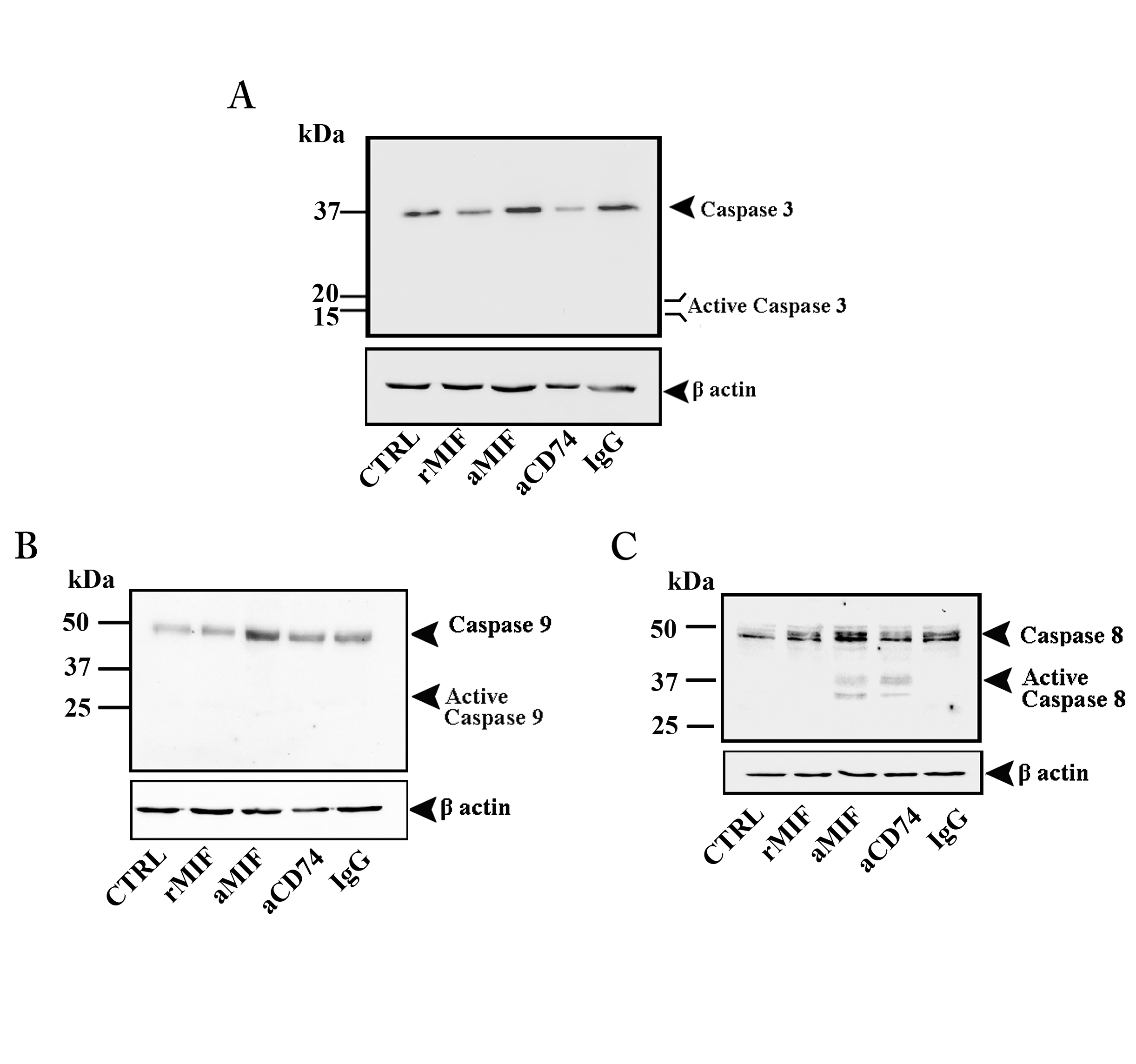


**Supplementary Figure 8S. Effect of inhibition of MIF and CD74 on placental apoptosis.** Representative western blots for caspase-3 **(A),**  caspase-9 **(B)** and caspase-8 **(C)** in explant cultures maintained at 20% O_2_. CTRL: control explant cultures**;** rMIF: recombinant MIF (100 ng/mL); aMIF: anti-human MIF antibody (10 µg/mL); aCD74: anti-human CD74 antibody (5 µg/mL); IgG: isotype matched IgG antibody (10 µg/mL). N=2 placentae (8 and 10 weeks).

**Original data**

***
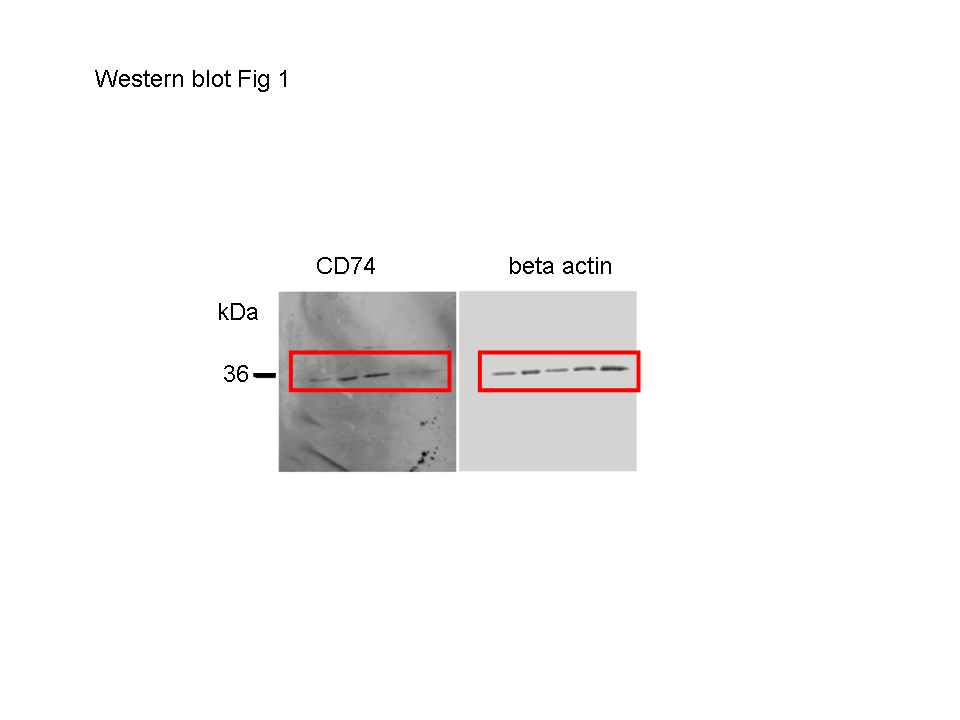
***

***
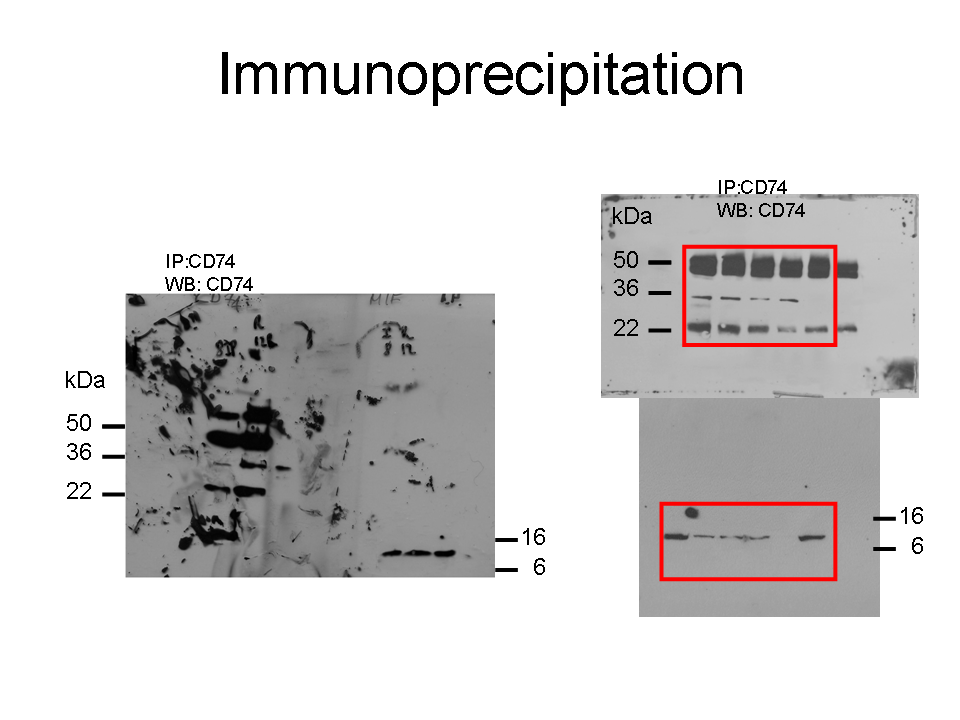
***

***Supplementary Information for Figure 1.*** Full-length blots of Figure 1B and 1E. *The red frame indicates the cropped area*


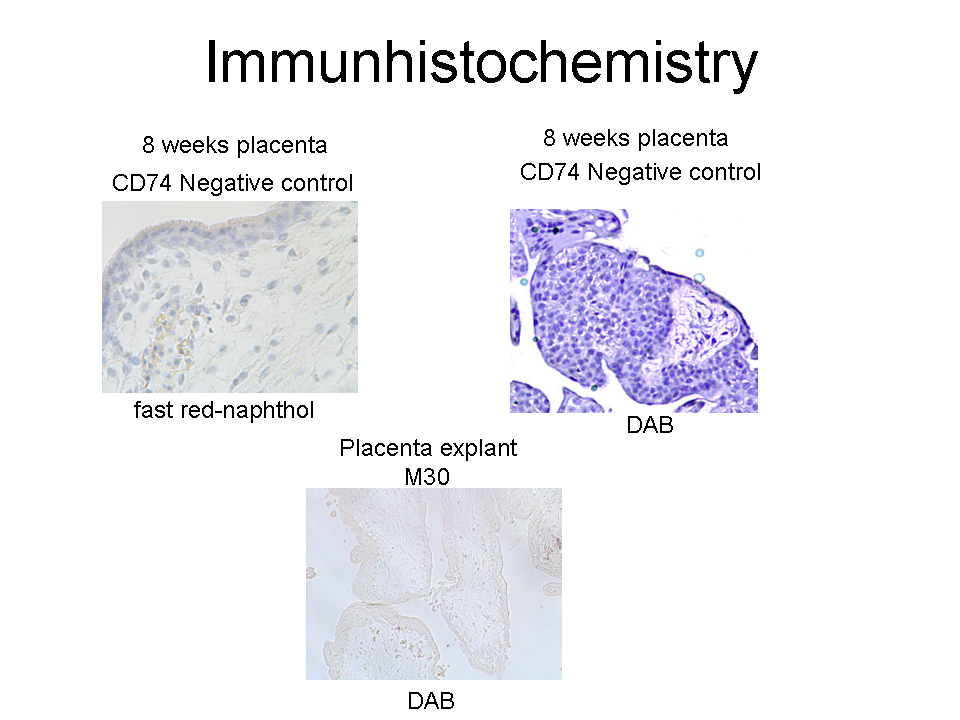


***Supplementary Information for Figure 1 and Figure 4****. Negative controls have been obtained by substituting the primary antibody with the appropriate normal isotype IgG.*


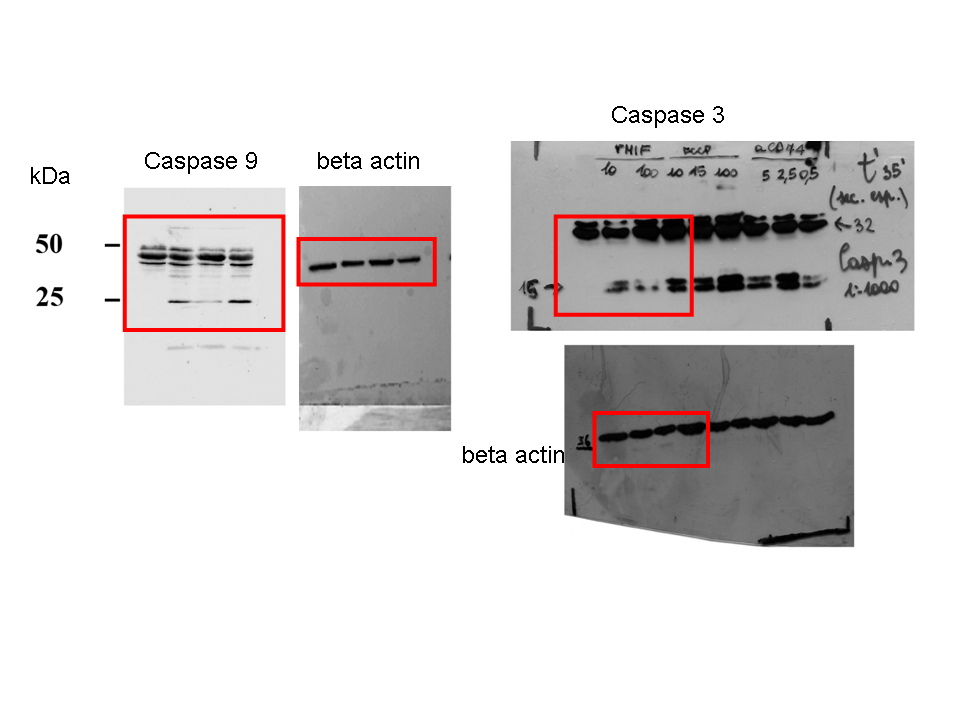


***Supplementary Information for Figure 3.*** Full-length blots of Figure 3A and 3C. *The red frame indicates the cropped area*


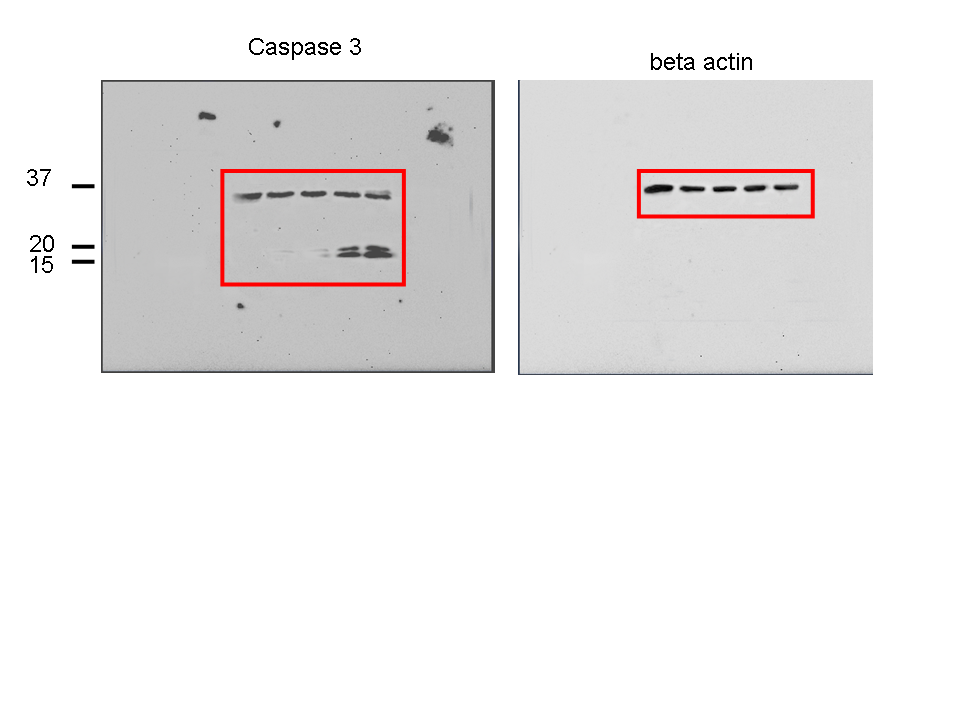


***Supplementary Information for Figure 4.*** Full-length blots of Figure 4A. *The red frame indicates the cropped area*


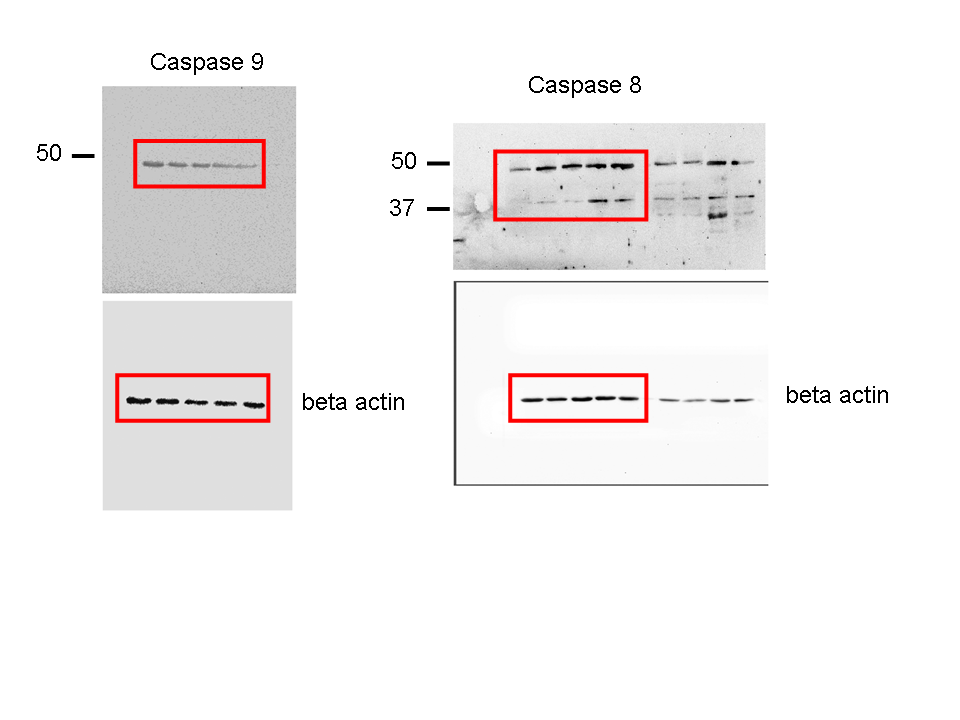


***Supplementary Information for Figure 5.*** Full-length blots of Figure 5A and 5B. *The red frame indicates the cropped area*


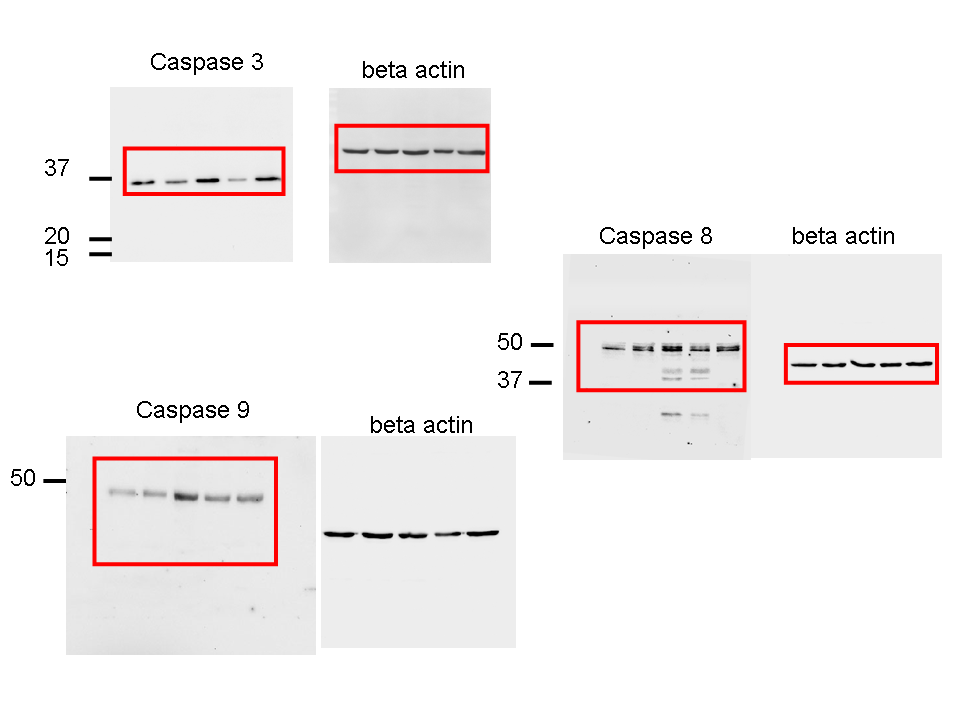


***Supplementary Information for Figure 8S.*** Full-length blots of Figure 5A and 5B. *The red frame indicates the cropped area*
